# The dynamic behaviour of metabolic syndrome and its components in an eight-year population-based cohort from the Mediterranean

**DOI:** 10.1371/journal.pone.0176665

**Published:** 2017-05-18

**Authors:** Maria A. Barceló, Antonio Rodríguez-Poncelas, Marc Saez, Gabriel Coll-de-Tuero

**Affiliations:** 1 Research Group on Statistics, Econometrics and Health (GRECS), University of Girona, Girona, Spain; 2 CIBER of Epidemiology and Public Health (CIBERESP), Madrid, Spain; 3 Research Support Unit. University Institute of Research in Primary Care Jordi Gol (IdIAP Gol), Girona, Spain; 4 Department of Medical Sciences, University of Girona, Girona, Spain; Medizinische Universitat Innsbruck, AUSTRIA

## Abstract

**Background:**

The significant rise in the prevalence of obesity coincides with the considerable increase in the prevalence of metabolic syndrome (MS) currently being observed worldwide. The components of MS are not static and their dynamics, such as the order of their occurrence, or the time of exposure to them are, as yet, unknown but could well be clinically relevant. Our objective was to study the dynamic behaviour of MS and its components in a large population-based cohort from a Mediterranean region.

**Methods and findings:**

Our study employed a retrospective cohort (between January 1, 2005 and December 31, 2012) made up of individuals from the general population in a region in the northeast of Catalonia, Spain. Given that most of the explicative variables of the risk of having MS were time dependent and, therefore, the risk was not proportional, we used the Andersen-Gill (AG) model to perform a multivariate survival analysis and inferences were performed using a Bayesian framework.

Thirty-nine percent of the participants developed MS; 44.6% of them with a single limited episode. Triglycerides and low HDL cholesterol, together with obesity, are components associated with the first occurrence of MS. Components related to the metabolism of glucose are associated with a medium risk of having a first episode of MS, and those related to blood pressure are associated with a lower risk. When the components related to blood pressure and the metabolism of glucose appear first, they determine the appearance of the first episode of MS. The variables concerning the persistence of MS are those that correspond to clinical conditions that do not have well-established drug treatment criteria.

**Conclusions:**

Our results suggest that the components related to the metabolism of glucose and to high blood pressure appear early on and act as biomarkers for predicting MS, while the components related to obesity and dyslipidaemia, although essential for the development of MS, appear later. Making lifestyle changes reduces the conditions associated with the persistence of MS.

## Introduction

Coinciding with the significant rise in obesity worldwide, a considerable increase in the prevalence of metabolic syndrome (MS) has been observed in recent times [1]. MS is defined as the co-occurrence of several cardiovascular risk factors in an individual. The most commonly-used definition is that of the NCEP ATP III [2,3], in which an individual is considered to have MS when diagnosed with at least three of the following five possible metabolic risk factors: i) abdominal obesity (waist circumference: men >102cm; women > 88cm), ii) impaired tolerance to glucose (fasting glucose levels ≥110 mg/dL or a diagnosis of type 2 diabetes mellitus, DM2), iii) high blood pressure (≥130/85 mmHg); iv) low high density lipoprotein cholesterol (HDL) (in men<40mg/dL; in women<50mg/dL), and v) hypertriglyceridemia (≥150 mg/dL) (NB: criteria iv and v are definers of dyslipidaemia).

MS has, in addition to its defining components [4], a number of predictive variables and, as such, being elderly, a woman [5], a smoker [6] or excessive alcohol consumption [7] have been associated with an increased risk of developing MS. Furthermore, most individuals with MS have some degree of insulin resistance. In fact, although the cause of MS is unknown, insulin resistance is considered to be responsible for many of the conditions that define MS, for instance, hyperglycaemia, hypertension, decreasing hepatic production of high density lipoproteins, increased triglycerides, and stimulation of endothelial profiling (which affects the endothelial receptors that trigger the onset of the atherosclerotic process) [8].

Nevertheless, MS components are not static and most are reversible with changes in lifestyle and/or pharmacological treatment [9,10]. However, little is known about the dynamic aspects of MS for example, the order in which its components occur, their exposure time, or the time that elapses between the occurrence of the first component and whether this time varies depending on which component materialises first [9,11–13].

In this paper, we use a large population-based cohort from a Mediterranean region to study not only the dynamic behaviour the different components of metabolic syndrome have, but also (once established) the metabolic syndrome itself.

## Methods

### Ethical considerations of the study

The data for this study came from an anonymised clinical administrative database and only the lead researcher, where necessary, had access to the identity of each individual. This study has also been revised and approved by the Ethics and Clinical Research Committee of the Institute of Health Care (IAS).

### Data setting

We used a (general population) retrospective cohort, composed of individuals, 15 years old or older who, between January 1, 2005 and December 31, 2012, had made use of the public primary healthcare services offered by one of the three Basic Areas of Health (ABS, acronym in Catalan) primary healthcare centres, which are managed by the Institute of Health Care (IAS, *‘Institut d’Assistència Sanitària’* in Catalan).

The IAS manages all the ABSs providing healthcare to the region known as ‘La Selva Interior’, Girona. According to the Catalan Institute of Statistics, in 2012 the region’s population constituted 32,860 men and 32,702 women (0.87% and 0.85%, respectively, of the entire Catalan population). The area is defined as a mainly rural (or semi-urban) territory. While there are a number of towns scattered throughout the area, it is also dispersed with farms, houses and small somewhat isolated villages. There are 144 municipalities in the region (representing 3.70% of Catalonia), but only five of them have more than 5000 inhabitants and only one has a little over 10,000. In 2012, the population density median was 85.5 hab/km^2^ and the average population density was 176.2 hab/km^2^ (compared to 235.8 hab/km^2^ in the whole of Catalonia [14].

Included in this study were adult individuals (15 years and older) who were registered with and had made use of the public primary healthcare service at one of the three ABS healthcare centres managed by the IAS during the study period.

Excluded from this study were: a) individuals who had sought medical care at one of the ABS healthcare centres managed by the IAS but were not registered with any of them (e.g. visiting population, emergencies etc), b) individuals with a previous diagnosis of arterial hypertension (HTA) or DM2 at the time the cohort was set up. Although we do not know the occurrence of (MS) components before the development of DM2 and/or HTA, these individuals had a high probability of having had an MS episode before the cohort had been formed. It is, therefore, quite likely that the probability of having an MS episode during the follow-up of the cohort was at least different, and probably higher, than in the case of the other individuals, thus causing a selection bias. In addition, these particular individuals, once they had been diagnosed with DM2 or HTA would have already started some kind of lifestyle change and/or pharmacological treatment, which could also imply a different probability of having MS.

A subject from the cohort was considered to have MS if they (simultaneously) exhibited three or more of the five possible conditions: i) a diagnosis of diabetes or impaired fasting glucose, ii) a diagnosis of hypertension or high blood pressure, iii) low high density lipoprotein levels (HDL), iv) hypertriglyceridemia, and/or v) obesity, BMI ≥ 30 kg/m^2^. We used this last criterion because we did not have any measurements of abdominal obesity. Obesity, in addition to being a defining criterion of MS (as specified by the WHO [15]), has always been closely correlated with an increased abdominal perimeter [16].

The criteria from the American Diabetes Association was used to diagnose DM2 [17]: clinical characteristics plus one random blood glucose level of ≥200 mg/dl, two basal glucose levels of ≥ 126 mg/dl, two determinations of HbA1c ≥ 6,5% or two 2-hour oral glucose tolerance tests ≥ 200 mg/dl. Subjects were also considered to have DM2 if they had been diagnosed with this condition previously or were receiving treatment with anti-hyperglycaemic drugs. High blood pressure was defined as having had at least two blood pressure readings taken in the doctor's surgery of between 130–139 and/or 85–89 mmHg. Subjects with at least two blood pressure readings of ≥140 and/or 90 mmHg, a previous diagnosis of hypertension or those receiving treatment with anti-hypertensive medication were considered to have hypertension.

As mentioned earlier, many of the MS components are dynamic. In fact, (although not the cases of diagnosed DM2 and diagnosed HTA), a subject who at one point fulfilled the defining conditions of a specific component (for example, high blood pressure), may subsequently stop complying with these conditions (i.e. the subject’s blood pressure readings return to normal), as a result of lifestyle change and/or pharmacological treatment. When we defined, as an episode of MS along with the period of time in which the subject met the defining conditions of MS, we ascertained three situations: first, there were (1) subjects who had more than one episode of MS during the follow-up period. Next, among the subjects who had only one episode of MS during the follow-up period, some of them (2) exited the MS episode (i.e. they did not meet DM defining conditions) before the end of the follow-up period (i.e. December 2012) and (3) others who, at the time of the end of the follow-up period, remained within the MS episode (i.e. they continued to meet its defining conditions). Thus, we now define situation 3 as being an episode of persistent MS and situations 1 and 2 episodes of non-persistent MS.

The start and finish dates for each of the (five) conditions were recorded. The dates when each episode of MS started and ended were also registered if the subject had more than one episode.

In addition to the variables used to define MS other variables were gathered during the follow-up including demographic variables (sex, date of birth and country of origin), main health complaints (summarised as the number of chronic illnesses), smoking and alcohol consumption, total cholesterol, creatinine, urinary albumin excretion, glomerular filtration rate, consumption of NSAIDS and treatment with anti-hypertensive, hypolipemiant or anti-diabetes drugs.

All the data were obtained from clinical records and stored following a standardized protocol in the (centralized) IAS information system. The data for this study were drawn from that information system conforming to an anonymized clinical-administrative database.

### Statistical methods

First, a description of the population included in this study was made. The qualitative variables were expressed as percentages, while the quantitative variables were expressed as mean and standard deviation and in the form of median and interquartile range.

Survival curves were estimated using the Kalbfleisch-Prentice method [18], which is equivalent to the Kaplan Meier estimates when the weights are unity (as in our case).

Given that a subject could have various episodes of MS and, with the exception of HTA and DM2, its defining components during the follow-up, and that all of the explicative variables—with the exception of sex and country of birth—vary over time, the risks are not proportional. For this reason, the Andersen-Gill model (AG) [19,20] of multivariant survival analysis was used [21]. The AG model allowed us to evaluate the multivariant survival and the time-dependent variables that could explain the risk of having MS. Apart from the design, with (multiple) dates for the start (and end) of episodes which varied for each individual, the AG model is characterised by introducing random effects that capture, at least, the fragility (also called non-observed individual heterogeneity) [21]. The observed confounding (both individual and contextual) was controlled by introducing the corresponding explicative variables into the models. With the control of the non-observed confusion, first we allowed the coefficients of some of the variables to be different for each individual and/or over time. In addition, we controlled the possibility of a long-term tendency non-parametrically (anticipating the possibility of changes in diagnostic procedures and/or treatment over the period under study, 2005–2012).

Given the complexity of our model, we preferred to perform inferences using a Bayesian framework. In particular, we followed the Integrated Nested Laplace Approximation (INLA) approach [22], within a (pure) Bayesian framework. All analyses were carried out with the free software R (version 3.2.2) [23], through the R INLA package [24,22].

## Results

### Descriptive results

**Fig 1:**
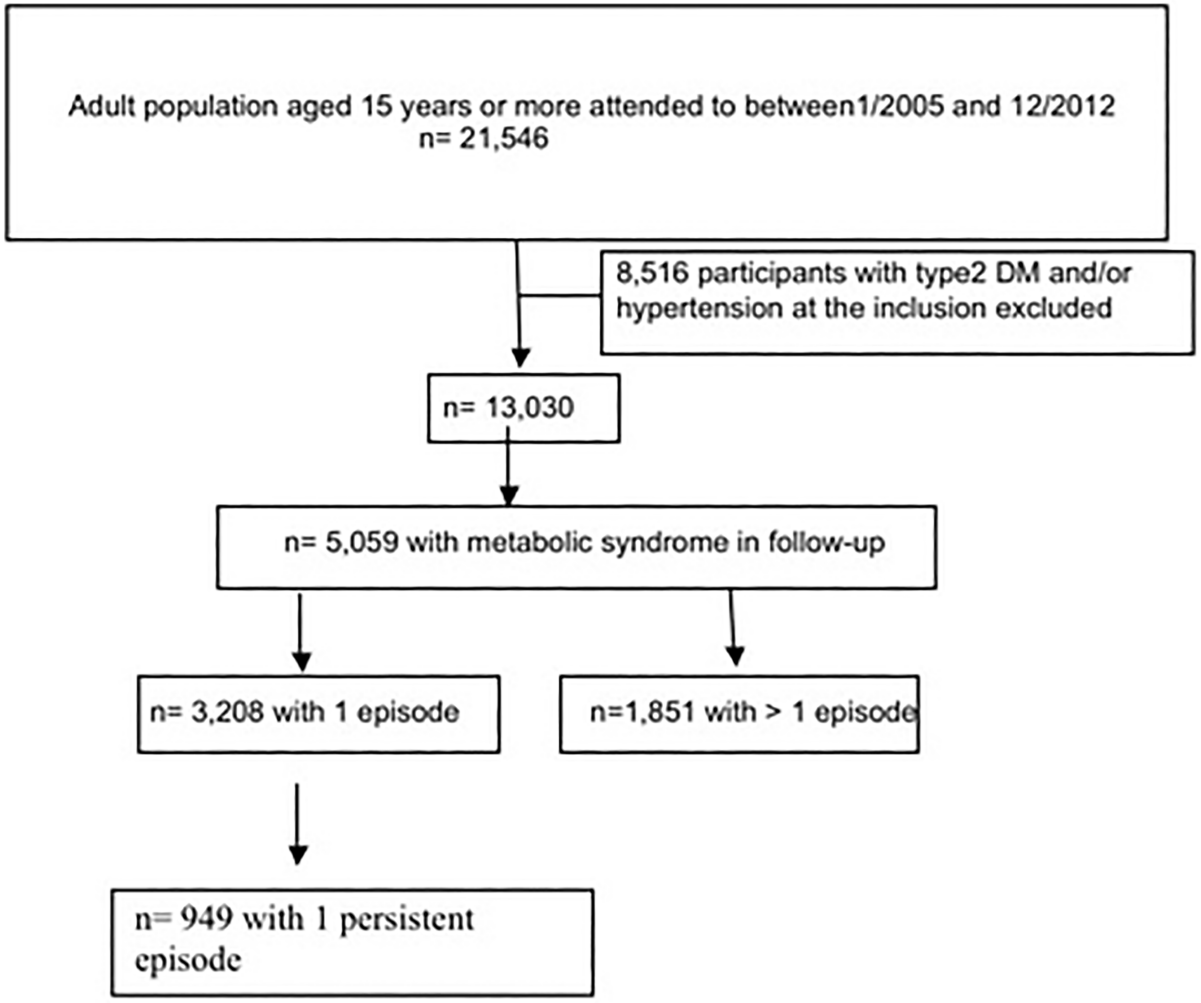
Flow-chart of cohort study.

The study consisted of 13,030 participants who met the inclusion criteria on 1^st^ January 2005. Fig 1 shows the flowchart of the cohort and Table 1 details the characteristics of the participants. The components of MS are distributed in the following way: 11.7% with BP ≥ 130/85 mmHg, 14.4% with impaired fasting glucose, 26.5% with dyslipidaemia and 38.6% of the subjects were obese. 51.8% had only one component of MS and 11.8% had two components.

**Table 1:** Baseline characteristics of cohort participants, 1/1/2005.

Age, years, mean (SD); median (p25-p75), [n of the sample]	52.11 (17.234); 52 (39–65) [20,727]
Gender, women, n (%), [n of the sample]	10.804 (52.1) [20,727]
BP, mmHg systolic, mean (SD); median (p25-p75), [n of the sample]	134.75 (17.97); 134 (123–144) [19.797]
BP, mmHg, diastolic, mean (SD); median (p25-p75), [n of the sample]	78.47 (11.11); 78 (71–86) [19,790]
BP high-normal 130-139/85-89 mmHg, Yes, n (%), [n of the sample]	2,310 (11.7) [19,788]
Hypertension, Yes, n (%), [n of the sample]	0 (0) [21,546]
DM2, Yes, n (%), [n of the sample]	0 (0) [21,546]
Fasting glucose, mg/dL, mean (SD); median (p25-p75), [n of the sample]	102.70 (89.91); 88 (81–99) [19,152]
Impaired fasting glucose, Yes,n (%), [n of the sample]	2.761 (14.4) [19,152]
Total Cholesterol, mg/dL, mean (SD); median (p25-p75), [n of the sample]	202.78 (41.87); 201.0(174.0–229.5) [19,189]
Hypercholesterolemia, Yes,n (%), [n of the sample]	9.774 (45.4) [21,546]
HDL-cholesterol;mg/dL. mean (SD); median (p25-p75), [n of the sample]	55.68 (15.69); 54 (45–65) [19,21]
Low- HDL cholesterol. Yes,n (%), [n of the sample]	4.513 (20.9) [21,546]
TGC mg/dL, mean (SD); median (p25-p75), [n of the sample]	127.46 (99.82); 104 (75–151) [18,576]
TGC ≥150 mg/dL, Yes, n (%), [n of the sample]	4.742 (22.0) [21,546]
Dyslipidaemia, Yes, n (%), [n of the sample]	5.712 (26.5) [21,546]
Creatinine,mg/dL. mean (SD); median (p25-p75), [n of the sample]	0.84 (0.23); 0.80 (0.70–0.94) [19,38]
Glomerular filtration rate, mL/min/1.73, mean (SD); Median (p25-p75)	50.60 (9.20); 53.6 (46.7–57.3) [2,344]
Urinary albumin creatinine ratio,mg/gr, mean(SD); median(p25-p75), [n sample]	19.75 (118.10); 5.00 (3.60–8.90) [10,750]
Microalbuminuria, Yes, n (%), [n of the sample]	870 (8.1) [10,750]
BMI, Kg/m2, mean, (SD); median (p25-p75), [n of the sample]	30.02 (5.40); 29.64 (26.35–33.01) [17,457]
Obesity (BMI≥30 Kg/m^2^), Yes, n (%), [n of the sample]	8,307 (38.6) [21,546]
MS 1 component only, Yes, n (%), [n of the sample]	11,151 (51.8) [21,546]
MS 2 components, Yes, n (%), [n of the sample]	2,546 (11.8) [21,546]
Any MS component, Yes, n(%), [n of the sample]	7,670 (35.6) [21,546]

n of the whole sample: 21,546.

BP: blood pressure; DM2: Type 2 diabetes mellitus; TGC: triglycerides; BMI: body mass index; MS: metabolic syndrome.

A total of 5,059 participants (38.8%) had some kind of episode of MS at some time during the follow-up: 3,208 (63.4%) had only one, either persistent or non-persistent, episode and 1,851 (36.6%) had more than one episode. By decreasing the (unadjusted) odds ratios, during follow-up, 47.7% of those with DM2 (compared to only 6.2% of those who did not), 49.4% of those with hypertension (only 7.3% of those who did not have HTA), 40.2% of those with dyslipidaemia (6.3% in those who did not), 42.2% of those with obesity (10% in non-obese participants), 55.7% of participants with impaired fasting glucose (compared to 18.9% of those who did not), and 35.3% of participants with high blood pressure (compared to 16.6% in those patients with had no high blood pressure), had some kind of episode of MS.

The basal characteristics of the patients with a single episode or more than one episode were similar in terms of gender, serum cholesterol and the percentage with low HDL cholesterol and BMI. Those with more than one episode of MS were older and were more likely to have impaired fasting glucose, dyslipidaemia and obesity (Table 2).

**Table 2:** Baseline characteristics of participants who developed one or more than one metabolic syndrome episode.

Variable	1 episode	>1 episode
Age, years, mean (SD); median (p25-p75)	54.59 (15.257); 54 (44–65)	57.45 (13.636); 58 (48–67)
Gender, women, n (%)	1,588 (49.5)	920 (49.7)
BP, mmHg Systolic, mean (SD); median (p25-p75)	135.70 (16.44); 135 (126–144)	136.43 (15.98); 135 (126–145)
BP, mmHg Diastolic, mean (SD); median(p25-p75)	79.16 (10.89); 79 (72–86)	79.6 (10.57); 78 (71–85)
BP high-normal 130-139/85-89 mmHg, Yes, n (%)	1,741 (53.6)	1,169 (62.4)
Fasting glucose, mg/dL (SD); median (p25-p75)	107.16 (53.56); 96.5 (88–112)	112.49 (58.27); 100 (89–119)
Impaired fasting glucose, Yes, n (%)	871 (26.8)	718 (38.3)
Total cholesterol mg/dL, mean (SD); median (p25-p75)	229.73 (49.19); 227 (198–258)	235.44 (42.23); 235 (206–261)
Hypercholesterolemia, Yes, n (%)	2,353 (73.8)	1,481 (79.4)
HDL cholesterol, mg/dL, mean (SD); median (p25-p75)	47.48 (13.15); 46 (38–55)	47.37 (11.43); 46 (39–53)
Low- HDL cholesterol, Yes, n (%)	1,795 (55.2)	1,084 (57.9)
TGC, mg/dL, mean (SD); median (p25-p75)	159.96 (124.00); 136 (96–186.25)	154.57 (74.85); 141 (104–185)
TGC ≥150 mg/dL, Yes, n (%)	2,018 (62.1)	1,318 (70.4)
Dyslipidaemia, Yes, n (%)	2,702 (83.1)	1,757 (93.8)
Creatinine, mg/dL, mean(SD); median (p25-p75)	0.943 (0.341); 0.90 (0.76–1.03)	0.976 (0.33); 0.91 (0.80–1.08)
Glomerular filtration rate, mL/min/1.73 m ^2^,mean (SD); Median (p25-p75)	52.49 (8.05); 55.3 (49.9–58.1)	53.91 (7.20); 56.4 (52.2–58.5)
Urinary albumin creatinine ratio, mg/gr, mean (SD); median (p25-p75)	38.97 (178.32); 7.4 (4.6–16.7)	42.86 (158.25); 8.2 (4.9–18.4)
Microalbuminuria, Yes, n (%)	365 (16.3)	283 (18.3)
BMI, Kg/m^2^, mean (SD); median (p25-p75)	29.62 (4.81); 29.34 (26.29–32.29)	29.78 (4.15); 29.40 (27.25–31.94)
Obesity (BMI≥30 Kg/m^2^), Yes, n (%)	2,236 (68.8)	1,660 (88.6)

BP: blood pressure; Impaired fasting glucose: ≥110 mg/Dl; HDL: high density lipoprotein; TGC: triglycerides; BMI: body mass index; MS: metabolic syndrome.

63.4% of the total number of participants who had MS had just one (persistent or non-persistent) episode, while 36.6% developed several episodes during the follow-up. Of the participants with a single episode, 70.4% had a non-persistent episode and 29.6% had a persistent episode. In comparison to the rest, the participants with a persistent episode of MS were younger, had higher percentages of obesity and dyslipidaemia, a lower percentage of high blood pressure and impaired fasting glucose and also urinary albumin excretion was lower. In other words, the opposite characteristics exhibited by subjects who had more than one episode of MS. Participants with a single non-persistent episode of MS were characterised as having a lower percentage of dyslipidaemia and obesity at the time of the first episode (Table 3). The combination of hypertension, obesity and impaired fasting glucose at the time of the first episode of MS was less frequent for participants with a single non-persistent episode of MS than for the rest of the group.

**Table 3:** Characteristics of participants at the moment of the first episode of metabolic syndrome, according to persistence or discontinuity.

Variable	1 episode Persistent MS	1 episode Non-persistent MS	> 1 episode
Age, years, mean (SD); median (p25-p75)	55.4 (16.32); 54 (44–67)	59.40(15.49); 59.5 (48–71)	62.34 (14.117); 63 (53–73)
Gender, women, n, (%)	465 (49.0)	1,123 (49.8)	920 (49.7)
BP high-normal 130-139/85-89 mmHg, Yes, n (%)	51 (5.3)	167 (7.3)	88 (4.7)
Hypertension, n (%)	610 (63.8)	1,776 (77.4)	1,774 (94.7)
Dyslipidaemia,n (%)	799 (83.6)	54 (2.4)	488 (26.1)
TGC ≥150 mg/dL, Yes, n (%)	590 (61.7)	26 (1.1)	311 (16.6)
Low HDL-cholesterol, Yes, n (%)	604 (63.2)	28 (1.2)	293 (15.6)
Glomerular filtration rate, mL/min/1.73m^2^mean (SD); median (p25-p75)	49.29 (9.14); 51 (44.05–57.1)	48.26 (10.95; 52.4 (42.55–56.7)	50.29 (9.50); 53.7 (46.5–57.2)
Urinary albumin creatinine ratio, mg/gr mean (SD); median (p25-p75)	19.28 (58.98); 5.2 (5–10.6)	29.02 (175.38); 5.3 (3.8–9.98)	22.08 (100.63); 5.1 (3.8–9.7)
Microalbuminuria, n (%)	60 (10.4)	164 (9.9)	143 (9.3)
BMI, Kg/m^2^ mean (SD); median (p25-p75)	30.49 (4.82); 30.22 (21.0–33.2)	29.29 (4.76); 28.93 (26.0–31.9)	29.78 (4.15); 29.40 (27.3–31.9)
Obesity (BMI≥30 Kg/m^2^), n (%)	480 (50.2)	184 (8.0)	524 (28.0)
Fasting glucose mg/dL mean (SD); median (p25-p75)	106.43 (46.56); 95 (87–110)	107.46 (56.23); 97 (88–112)	112.49 (58.27); 100 (89–119)
Impaired fasting glucose, n (%)	87 (9.1)	300 (13.1)	293 (15.6)
Hypertension+Obesity+IFG, n (%)	122 (9.3)	566 (20.7%)	382 (35.2%)
Hypertension+Obesity+ Dyslipidaemia, n(%)	587 (44.8)	1283 (47.0)	850 (78.4)
Hypertension+IFG+ Dyslipidaemia, n(%)	644 (49.2)	1,603 (58.7)	934 (86.2)
Hypertension+Type 2 DM2+Obesity, n (%)	757 (57.8)	1,862 (68.2)	982 (90.6)
Hypertension+Type 2 DM2+ Dyslipidaemia, n (%)	927 (70.8)	2,253 (82.5)	1,044 (96.3)
High-normal BP+Obesity+IFG, n (%)	927 (70.9)	2,264 (82.9)	1,045 (96.4)
High-normal+BP+Obesity+ Dyslipidaemia, n (%)	957 (73.1)	2,445 (89.5)	1,069 (98.6)
High-normal BP+IFG+ Dyslipidaemia, n (%)	960 (73.3)	2,452 (89.8)	1,071 (98.8)
High-normal BP+DM2+Obesity, n (%)	966 (73.8)	2,478 (90.7)	1,071 (98.8)
High-normal BP+DM2+ Dyslipidaemia, n (%)	978 (74.7)	2,506 (91.8)	1,072 (98.9)

BP: blood pressure; IFG: impaired fasting glucose ≥110 mg/dL: TGC: triglycerides; HDL-cholesterol: high density lipoprotein; BMI: body mass index; MS: metabolic syndrome; Dyslipidaemia: TGC ≥150 mg/dL and/or low HDL-cholesterol, Microalbuminuria: urinary albumin creatinine ratio ≥ 30 mg/gr.

### Results of the multivariate analyses

The multivariate analysis (AG) (Table 4) showed that women had a lower risk of a first episode of MS (HR 0.94; 95% CI 0.84–0.99; p<0.01). After the age of 75 the risk of having a first episode of MS increased (HR 1.20; 0.97–1.48; p = 0.04) and smoking and alcohol consumption also posed an increased risk (HR 1.28; 1.06–1.54; both). All of the components of MS were related to some degree to the appearance of a first episode of MS. Triglycerides ≥150 mg/dL (HR 2.84; 2.68–3.00), obesity (HR 2.71; 2.57–2.85) and low HDL-cholesterol (HR 2.47; 2.33–2.62) were the components that were most closely related to the first episode of MS.

**Table 4:** Variables related to the risk of the occurrence of the first episode of metabolic syndrome and to the risk of the occurrence of any episode of metabolic syndrome. Multivariate analyses.

Variables	First episode		Any episode	
HR (95% CI)	HR	95% CI	HR	95% CI
Gender (Men) Women	0.94	0.84–0.99	0.94	0.90–0.98
Age (<40 years) 40–44	0.99	0.81–1.22	1.20	0.98–1.47
45–49	0.91	0.74–1.12	1.19	0.97–1.44
50–54	1.07	0.87–1.30	1.22	1.00–1.48
55–59	1.04	0.85–1.28	1.23	1.01–1.49
60–64	1.11	0.91–1.36	1.28	1.06–1.55
65–69	1.13	0.92–1.39	1.25	1.03–1.51
70–74	1.01	0.82–1.25	1.23	1.01–1.50
75–79	1.20	0.97–1.48	1.19	0.98–1.46
80–84	1.19	0.95–1.49	1.19	0.97–1.47
≥ 85	1.68	1.32–2.14	1.42	1.13–1.78
Triglycerides ≥150 mg/dL	2.84	2.68–3.00	2.06	1.96–2.16
Obesity (BMI≥30 Kg/m^2^)	2.71	2.57–2.85	2.50	2.39–2.61
Low HDL-cholesterol	2.47	2.33–2.62	1.75	1.67–1.84
DM2	1.72	1.61–1.84	1.00	0.94–1.07
Impaired fasting glucose	1.67	1.49–1.88	1.42	1.26–1.59
Hypertension	1.56	1.46–1.66	1.04	0.98–1.10
High-normal BP 130-139/85-89 mmHg	1.31	1.17–1.46	1.46	1.31–1.62
Smoking (never) Yes	1.13	1.04–1.22	0.98	0.91–1.05
Ex-smoker	0.93	0.81–1.05	0.97	0.86–1.10
Alcohol (no) Yes	1.28	1.06–1.54	0.97	0.83–1.13
Ex-consumer	0.90	0.70–1.17	0.97	0.77–1.23

CI: credibility interval; HDL: high density lipoprotein; DM2;type 2 dabetes mellitus;BP: blood pressure; Impaired fasting glucose: ≥ 110 mg/dL.

Adjusted for: country of origin (Spain, other), number of chronic diseases, antihypertensive drug, antidiabetic drugs, hypolipemiant drugs and non steroidal anti-inflammatory drugs.

Age was also associated with a higher risk of one episode of MS, rising from the age of 40 years onwards (HR 1.20;0.98–1.47; p = 0.003). Women had a lower risk of having more than one episode (HR 0.94;0.90–0.98). Smoking and alcohol consumption did not increase the risk of having more than one episode of the condition. Obesity was the component associated with the highest risk of developing more than one episode of MS (HR 2.50; 2.40–2.60). Type 2 diabetes (DM2) and hypertension were not associated with a higher risk of having more than one episode.

The multivariate analysis was also performed for premorbid metabolic syndrome (without DM2) and the results were comparable with those of general MS (data not shown).

**Fig 2:**
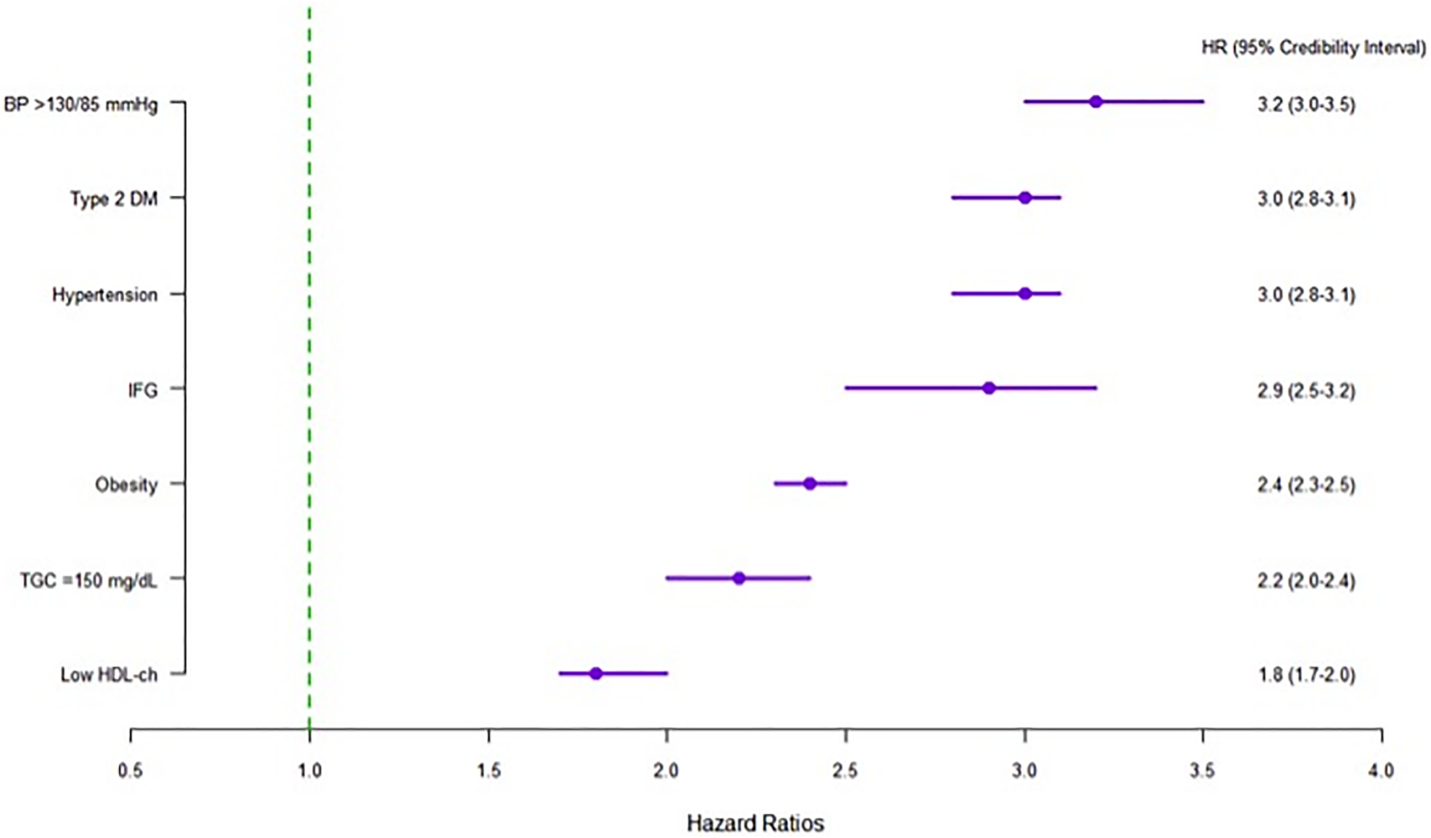
Order in which the components appear and risk first metabolic syndrome episode.

High-normal BP (HR 3.20; 3.00–3.50), DM2 and hypertension appearing first (HR 3.00; 2.80–3.10; both) were associated with a higher risk of developing a first episode of MS during the follow-up, whereas when the components related to dyslipidaemia appeared first, this was associated with a lower risk of experiencing a first episode of MS (low HDL cholesterol- HR 1.80; 1.70–2.00- and TGC ≥150 mg/dL-HR 2.20; 2.00–2.40) (Fig 2).

**Fig 3:**
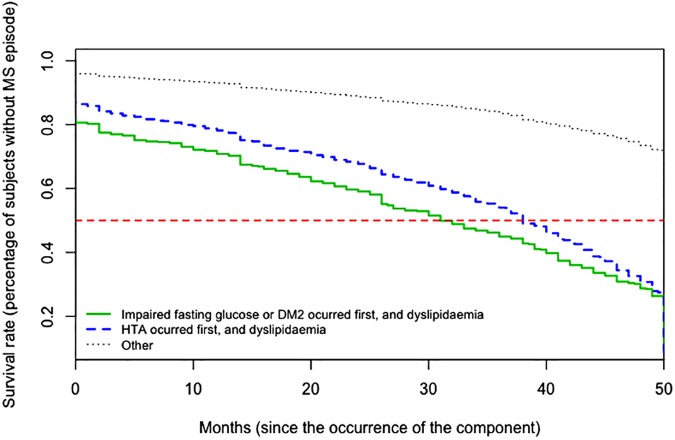
Survival curves. **Survival rate (percentage of subjects without MS episode) from the occurrence of the component, in accordance with the first occurrence of components related to glucose metabolism, combined with dyslipidaemia.** The horizontal line represents a 50% probability (of the first MS episode occurring).

While there are other possible combinations of components that are first to appear, when those related to the metabolism of glucose (impaired fasting glucose and DM2) or hypertension are combined with dyslipidaemia (TGC ≥ 150 mg/dL or low HDL cholesterol), and are the first to appear, the risk of the initial episode of MS is at its highest (HR ≥ 2.00) (Table 5). In other words, when the components related to the metabolism of glucose occurred first and the subject also had dyslipidaemia, there was more than a 50% likelihood of the first episode of MS occurring at 32 months (from the beginning of the follow-up), while if the first occurrence was hypertension (combined with dyslipidaemia), a more than 50% probability of an initial MS episode occurring, transpires at 38 months. In the other cases, the probability is not more than 50% likely until 50 months (Fig 3).

**Table 5:** Risk of the first episode of metabolic syndrome in relation to the occurrence of the first two components. Multivariate analysis.

	DM2	IFG	TGC≥ 150 mg/dL	LowHDL-ch	HT	High-normal BP	Obesity BMI ≥ 30 Kg/m^2^
**DM2**	x	NA	3.3 (2.6–4.1)	2.6 (2.0–3.4)	1.4 (1.2–1.6)	0.9 (0.7–1.2)	1.5 (1.3–1.7)
	**IFG**	x	2.0 (1.5–2.7)	1.6 (1.1–2.4)	0.9 (0.6–1.5)	0.9 (0.7–1.3)	1.6 (1.3–2.1)
		**TGC≥150 mg/dL**	x	1.7 (1.6–1.9)	2.0 (1.6–2.5)	1.7 (1.3–2.3)	1.8 (1.4–2.3)
			**LowHDL-ch**	x	2.1 (1.7–2.6)	1.0 (0.7–1.4)	1.6 (1.2–2.1)
				**HT**	x	NA	1.2 (1.1–1.4)
					**BP ≥130/85 mmHg**	x	1.9 (1.5–2.4)

IFG: impaired fasting glucose; TGC: triglycerides; HT: hypertension; BP: blood pressure; High-normal BP: blood pressure between 130–139 and/or 85–89 mmHg; BMI: body mass index; IFG: Impaired fasting glucose: ≥ 110 mg/Dl; N/A: not applicable.

Data are expressed as Hazard ratio and 95% Credibility Interval.

Adjusted for age, country of origin (Spain, others), number of chronic diseases, tobacco, alcohol, antihypertensive drugs, antidiabetic drugs, hypolipemiant drugs and non-steroidal anti-inflammatory drugs.

In red: HR ≥ 2.0; in yellow: HR < 2.0 (significant); in green: HR ≤ 1.0 (no significant).

### Persistence of MS episode

Table 6 shows the variables related to the occurrence of a single persistent episode of MS and to one or more episodes of non-persistent MS. Age, high-normal BP, triglycerides ≥ 150 mg/dL, low HDL cholesterol and impaired fasting glucose were significantly associated and were independent of the persistency of MS. Hypertension and DM2, on the other hand, were the variables that were most strongly associated with the non-persistence of MS. Age was associated with the non-persistence of MS from 25 years onwards and this association was stronger as age increased. No differences were found between one non-persistent episode and more than one non-persistent episode. Impaired fasting glucose, smoking and alcohol consumption were associated with both the persistence and the non-persistence of MS, in other words they were associated with having MS in general.

**Table 6:** Variables related to the risk of persistent or non-persistent metabolic syndrome. Multivariate analysis.

Type of episode	1 persistent episode		1 non-persistent episode		>1 non-persistent episode	
HR 95% CI	HR	95% CI	HR	95% CI	HR	95% CI
Gender [men] Women	0.99	0.96–1.02	0.87	0.81–0.95	0.87	0.81–0.95
Age [<25 years], 25–29	1.01	0.71–1.44	1.38	1.03–1.86	1.38	1.03–1.86
30–34	1.03	0.64–1.39	2.06	1.58–2.70	2.06	1.58–2.70
35–39	1.04	0.79–1.25	2.60	2.01–3.37	2.60	2.01–3.36
40–44	1.08	0.99–1.17	3.10	2.41–3.98	3.09	2.40–3.90
45–49	1.15	1.06–1.24	3.57	2.79–4.57	3.56	2.78–4.56
50–54	1.28	1.19–1.39	4.49	3.51–5.74	4.47	3.50–5.72
55–59	1.40	1.30–1.52	4.81	3.77–6.16	4.80	3.76–6.13
60–64	1.51	1.39–1.63	5.46	4.27–6.95	5.44	4.26–6.96
65–69	1.50	1.39–1.63	5.72	4.47–7.33	5.70	4.45–7.30
70–74	1.63	1.50–1.76	5.16	4.02–6.62	5.13	4.00–6.59
75–79	1.82	1.68–1.98	5.31	4.12–6.85	5.29	4.10–6.80
80–84	2.11	1.93–3.31	6.00	4.61–7.82	5.97	4.59–7.77
≥85	2.45	2.21–2.73	7.05	5.29–9.40	7.00	5.26–9.35
Triglycerides≥150 mg/dL	1.14	1.09–1.19	0.98	0.92–1.04	0.98	0.92–1.04
Obesity (BMI≥30 Kg/m^2^)	0.72	0.60–0.72	0.97	0.93–1.01	0.97	0.93–1.01
Low HDL-cholesterol	1.32	1.27–1.38	0.94	0.87–1.01	0.94	0.87–1.01
DM2	0.81	0.78–0.84	2.20	2.09–2.35	2.22	2.10–2.35
Impaired fasting glucose	1.25	1.16–1.35	1.33	1.19–1.49	1.33	1.19–1.49
Hypertension	0.80	0.76–0.83	2.14	2.01–2.27	2.15	2.02–2.28
High-normal BP	2.92	2.81–3.03	1.57	1.45–1.70	1.57	1.45–1.70
Smoking [never] Yes	1.37	1.31–1.43	1.39	1.27–1.53	1.39	1.26–1.52
Ex-smoker	0.73	0.67–0.80	1.07	0.92–1.25	1.07	0.92–1.25
Alcohol [no] Yes	1.21	1.08–1.36	1.40	1.14–1.72	1.40	1.14–1.72
Ex-consumer	0.76	0.62–0.92	0.94	0.68–1.29	0.94	0.68–1.29

Reference category in square brackets.

CI: credibility interval; High-normal BP blood pressure: blood pressure between 130-139/and/or 85–89 mmHg; Impaired fasting glucose: ≥ 110 mg/dL; HDL: high density lipoprotein; DM2: type 2 diabetes mellitus.

Adjusted for: country of origin (Spain, other), number of chronic diseases, antihypertensive drugs, antidiabetic drugs, hypolipemiant drugs and non-steroidal anti-inflammatory drugs.

## Discussion

In summary, our main findings can be summarized as follows:

- During the follow-up over a third of the participants (39%) developed MS, with the large majority of these having a single limited episode (non persistent) (44.6%).
- We found that triglycerides, low HDL cholesterol and obesity were the best predictors of the first episode of MS. The components related to the metabolism of glucose were associated with a medium risk of developing a first episode of MS. Those related to blood pressure were associated with a lower risk.
- The components related to blood pressure and the metabolism of glucose—high-normal BP, DM2, hypertension and impaired fasting glucose—were, in this order, those that, when they appear first, determine having a first episode of MS, whereas the components related to dyslipidaemia were associated with a lower risk when they appear first.
- The variables related to the persistence of MS are what take centre stage, as they correspond to clinical conditions that do not have well-established drug treatment criteria (triglycerides ≥150 mg/dL, low HDL cholesterol, impaired fasting glucose and high-normal BP).

### Occurrence of MS

The 8-year follow-up of the general population-based cohort who had sought medical care in public primary healthcare centres shows that over a third of the participants (39%) developed MS, with the large majority of these having a single limited episode (non persistent) (44.6%). While age is associated with a higher risk of having MS, women have a lower risk of developing the condition than men do. The incidence found in this study is similar to that described in a 10-year cohort from the Middle East, which showed a 44% occurrence of MS [13]. The MS prevalence described oscillates between 19.6% in the Mediterranean population [25] and 34.7% in the Middle Eastern population [26]. A study recently carried out in Spain found a 31% prevalence [27]. The differences observed can probably be explained by the different designs of the studies in question and the criteria they used to define MS. As other authors have concluded, smoking and alcohol consumption are associated with a higher risk of MS [6,7].

There is an enormous variation between countries in the prevalence of MS and depends on the demographic structure of the population. In our study, we found that women had a lower risk of a first episode of MS. This could be because, in our case, the population is younger than in other studies and in young populations the prevalence of MS is higher among men than among women. These results are also found by other studies, both in populations similar to [28] and more remote from ours [29, 30]. For the case of Spain, Martínez-Larrad et al., found that there was a 34.2% prevalence of MS in men and 28.5% in women [28]. Scuteri et al., after a 6-year follow-up using the Baltimore Longitudinal Study on Aging (BLSA), estimated an incidence of MS of (on average) 25.5% in men and 14.8% in women [29]. In the Chinese population, Chen et al., also found that men were more prone to developing MS than women of the same age, and that increasing age was an important factor in inducing MS [30].

### Predictors of the risk of MS

In this study triglycerides and low HDL cholesterol are the best predictors of the first episode of MS, along with obesity, while the components related to the metabolism of glucose are associated with a medium risk of developing a first episode of MS and those related to blood pressure are associated with a lower risk. These data concur with those of the cohort from the Middle East [13].

### The dynamics of MS

The sequence in which the components of MS appear, determines a higher risk of having a first, or more than one, episode of MS. The components related to blood pressure and the metabolism of glucose—high-normal BP, DM2, hypertension and impaired fasting glucose—are, in this order, those that, when they appear first, determine having a first episode of MS, whereas the components related to dyslipidaemia are associated with a lower risk when they appear first. A recent study showed that individuals with normal-high blood pressure have a higher risk of developing metabolic disorders and MS compared to those with normal blood-pressure [31]. In line with our work, three other studies have examined the dynamic behaviour of risk factors for metabolic syndrome [30,32,33] and, although they refer to different populations and use other methods of analysis, their findings are very close to ours. Haring et al., identified low HDL as the risk factor most associated with the onset of MS, and hypertension and central obesity were the predominant risk factors [32]. Hwang et al., point out that individuals with one or two components showed increased development of MS [33]. We have found just one paper, carried out on a Chinese population, also analysing the influence of the order in which the components of MS appear [30]. The results were similar to ours and showed that glucose intolerance in individuals under 40 years old and normal-high blood pressure in those over 40 years old are the components that, when they appear first, determine a higher risk of developing MS.

The results indicate that the components related to a higher risk of MS when they appear first do not coincide with the components whose presence at any time during the follow-up are also associated with this risk. It would seem that the components related to the metabolism of glucose and blood pressure appear early on and act as biomarkers for the evolution of MS, and while the components related to obesity and dyslipidaemia may appear later their presence is essential for the development of MS. Supporting this hypothesis are the data shown in Table 6 that demonstrate how patients with a higher risk of a first episode of MS are those whose first two components to appear are one related to blood pressure plus another related to dyslipidaemia. Furthermore, the combination of these components, when they are the first to appear, is associated with a low or nil risk of developing MS. It is likely, as mentioned earlier, that the function of risk biomarker is not synergistic between the two components but that these components independently (and at an early stage) express the metabolic vulnerability of the patient. Accordingly, high blood pressure and impaired glucose metabolism make the early detection of subjects with a very high risk of developing MS possible, allowing them to be prioritised for making the necessary lifestyle changes for both these components and for the later appearance of obesity and dyslipidaemia. Physical exercise [34,35] and changes in diet [36] have been shown to not only reduce the components of MS, but also to prevent its occurrence. This same reasoning may also explain why subjects' triglycerides may experience a substantial change, even though their HDL levels remain stable during the study follow-up period because these participants may have already adjusted their habits towards healthier lifestyles by increasing physical activity and/or changing their diet.

### Biological plausibility

It is difficult to establish which particular metabolic component leads to the cascade of disorders characterizing the MS syndrome, but individuals with an isolated hyperglycaemia state have a greater prospect of developing MS. There are some overlapping metabolic pathways in the pathogenesis of the four components of MS. Hyperglycaemia could lead to obesity, and central obesity has been confirmed to be the most relevant predisposing factor for insulin resistance. Diabetes is often accompanied by hypertension and these two conditions share conjunct pathways such as the renin-angiotensin aldosterone system, the sympathetic nervous system, adipokines, the inflammatory pathway, and oxidative stress. Glycaemic control and high blood pressure in particular, ought to attract more attention and that once individuals have been diagnosed with hyperglycaemia and high blood pressure, they should receive active defence measures to prevent the other components of MS emerging. Previous studies have revealed that people with normal glycaemia and optimal and normal blood pressure were less susceptible to developing MS [37].

The variables related to the persistence of MS are what take centre stage, as they correspond to clinical conditions that do not have well-established drug treatment criteria (triglycerides ≥150 mg/dL, low HDL cholesterol, impaired fasting glucose and high-normal BP). However, these clinical conditions can be improved through lifestyle intervention, especially in terms of physical exercise and weight loss. Meanwhile, obesity, DM2 and hypertension do not show any association with the persistence of MS and a possible explanation for this is that when a person is diagnosed with DM2, obesity or hypertension a non-pharmacological approach is always initiated as well as, in most cases, a drug treatment for the clinical condition and the associated risk factors, many of which are components of MS. Controlling these components means that the patient no longer presents the criteria for the diagnosis of MS. Currently, information on the importance of the time of exposure to MS is scarce [38], although we must assume that there is a direct relationship between the two.

### Limitations

This study has various limitations. First, the fact that is was a retrospective cohort meant that information about the waist circumference of many of the individuals was not available. Consequently, obesity (BMI ≥ 30 Kg/m^2^) was considered to be a component of MS instead. However, almost all of the individuals above this value meet the defined criteria for abdominal obesity [16] and that, second, BMI is a good predictor of the risk of MS [39], CV mortality and morbidity and all-cause mortality [40].

A second limitation is that patients were observed only when they had contact with the primary care services, so the beginning and end of an MS episode were not known, only their lower and upper limits.

A third limitation was a threat of selection bias. Some individuals could have a higher probability of having used primary health care, implying that the potential result, 'contact registration', was overrepresented in the sample observed [41]. However, in the first three years of the follow-up of the cohort, 78.33% of the assigned adult population (15 years and older), had contact with primary care services managed by the IAS, and throughout the entire follow-up (2005–2012) 94.41% of the population had contact with these services. Nevertheless, and again, as a robustness check, we repeated the analyses using a selection bias-free method proposed by Saez *et al*. [41]. As expected, the results did not change significantly.

Our final limitation was the presence of missing observations in most of the variables of interest. However, we performed a sensitivity analysis (see Appendix 1: Statistical Annex) and found that the missing data could not be associated with any of the variables analysed, implying that there was no selection bias.

## Conclusions

In summary, the data here show that more than a third of the cohort developed MS during the 8-year follow-up, with 44% of these subjects having one limited episode (non-persistent). The components related to blood pressure and the metabolism of glucose are the early predictors of the risk of developing the first episode of MS, but their later association with the components of dyslipidaemia is crucial (TGC≥150 mg/dL or low HDL cholesterol). As not all individuals with MS enter the syndrome with an identical combination of factors, certain trajectories and combinations of components confer higher risks, among which hyperglycaemia and high blood pressure conferred a significantly higher risk when compared with the others. These results could allow us to identify members of the population with a very high risk of developing a first episode of MS. In particular, not only should every effort to identify individuals with this particular combination (i.e. hyperglycaemia and high blood pressure) be made, but so should every effort be made to provide them with adequate treatment at early stages of disease. Once these individuals have been identified, their GP should priorize the lifestyle changes each MS component requires, above all weight control and physical exercise which could prevent the occurrence of MS.

That said, we may still have some unresolved issues. For instance, we do not know whether the cardiovascular risk associated with MS corresponds to the sum of the risk of its components or, are there perhaps synergistic effects between them that make the risk greater? Furthermore, other areas of interest in metabolic syndrome research include non-alcoholic fatty liver disease and liver steatosis, age and gender specific profiles and the possible ways for treatment optimization and these are all aspects that, undoubtedly, deserve future research efforts.

## Appendix 1.- Statistical annex. Sensitivity analyses

We assumed that the observations missing were completely at random (MCAR), that is to say, that the probability of an observation missing did not depend on observed or unobserved measurements. As a robustness check, we tested this using estimated logistic regressions, with the response variable being the presence (or not) of a missing observation in MS and in each one of its components and explanatory variables, sex, age, the municipality of the individual’s place of residence, the individual’s country of birth and the month and year of the incidence of the missing data. None of these explanatory variables were associated with the presence of the missing data in the variables of interest.

As in any Bayesian analysis, the choice of the prior distributions of model parameters (i.e. priors) may have had a considerable impact on the results. However, we used priors that penalize the complexity (PC priors) [42] and which have been found to be very robust. Furthermore, we performed sensitivity analyses to assess how the prior on the hyperparameters influenced the estimation results. First, by increasing the precision (lowering the variance) and second, by testing other priors, those used by default in R INLA (log gamma) with different shape and inverse-scales; uniform and centred half-normal. In all cases the PC priors provided better results.

## Abbreviations

ABS: *‘Àrea Bàsica de Salut’* Basic Health Areas
AG: Andersen-Gill model
BMI: Body Mass Index
BP: Blood pressure
CV: Cardiovascular
CVR: Cardiovascular risk
DM2: Type 2 diabetes mellitus
HbA1c: Glycated haemoglobin
HDL: High density lipoproteins
HR: Hazard rate
HTA: Hypertension
IAS: *‘Institut d’Assistència Sanitària’*, Health Care Institute
INLA: Integrated Nested Laplace Approximation
MCAR: Missing completely at random
MS: Metabolic syndrome
NCEP ATP III: Third Report from the National Cholesterol Education Program
NSAIDS: Non-steroidal anti-inflammatory drugs
PC priors: Penalizing complexity priors
TGC: Triglycerides
WHO: World Health Organization
